# Higher Expression of Annexin A2 in Metastatic Bladder Urothelial Carcinoma Promotes Migration and Invasion

**DOI:** 10.3390/cancers14225664

**Published:** 2022-11-18

**Authors:** Christina Guo, Rucha Trivedi, Amit K. Tripathi, Rajesh R. Nandy, Diana C. Wagner, Kalyani Narra, Pankaj Chaudhary

**Affiliations:** 1 Texas College of Osteopathic Medicine, University of North Texas Health Science Center, Fort Worth, TX 76107, USA; 2 Department of Microbiology, Immunology and Genetics, School of Biomedical Sciences, University of North Texas Health Science Center, Fort Worth, TX 76107, USA; 3 Department of Biostatistics and Epidemiology, School of Public Health, University of North Texas Health Science Center, Fort Worth, TX 76107, USA; 4 Department of Anatomic Pathology, JPS Health Network, Fort Worth, TX 76104, USA; 5 JPS Oncology and Infusion Center, JPS Health Network, Fort Worth, TX 76104, USA

**Keywords:** bladder urothelial carcinoma, Annexin A2, plasmin, migration, invasion

## Abstract

**Simple Summary:**

Bladder urothelial carcinoma (BLCA) arises from basal cells that develop dysplasia and carcinoma in situ and, if unchecked, progress to invasion and metastases. Major players underlying the molecular mechanisms of invasive and metastatic BLCA are yet to be determined. Annexin A2 (AnxA2), a Ca^++^-dependent phospholipid-binding protein, is overexpressed in various cancers and facilitates cell migration and invasion. This study established a correlation between AnxA2 and BLCA using existing BLCA data from the Cancer Genome Atlas (TCGA). In addition, we determined its role in the proliferation, migration, and invasion of metastatic bladder cancer cells.

**Abstract:**

In this study, we aim to evaluate the significance of AnxA2 in BLCA and establish its metastatic role in bladder cancer cells. Analysis of TCGA data showed that AnxA2 mRNA expression was significantly higher in BLCA tumors than in normal bladder tissues. High mRNA expression of AnxA2 in BLCA was significantly associated with high pathological grades and stages, non-papillary tumor histology, and poor overall survival (OS), progression-free survival (PFS), and diseases specific survival (DSS). Similarly, we found that AnxA2 expression was higher in bladder cancer cells derived from high-grade metastatic carcinoma than in cells derived from low-grade urothelial carcinoma. AnxA2 expression significantly mobilized to the surface of highly metastatic bladder cancer cells compared to cells derived from low-grade tumors and associated with high plasmin generation and AnxA2 secretion. In addition, the downregulation of AnxA2 cells significantly inhibited the proliferation, migration, and invasion in bladder cancer along with the reduction in proangiogenic factors and cytokines such as PDGF-BB, ANGPT1, ANGPT2, Tie-2, bFGF, GRO, IL-6, IL-8, and MMP-9. These findings suggest that AnxA2 could be a promising biomarker and therapeutic target for high-grade BLCA.

## 1. Introduction

Bladder cancer is the 6th most common cancer in the US, with 81,180 estimated new cases and 17,100 estimated deaths in 2022 [1,2]. More than 90% of these cases are bladder urothelial carcinoma (BLCA). The prognosis and treatment options for BLCA are highly variable. Non-muscle invasive bladder cancers include stages 0 and 1. They comprise non-invasive papillary Ta, in situ Tis, tumors that invade lamina propria T1 [3]. These make up about 70% of all bladder cancers, are treated with localized approaches, and have an overall good prognosis but recur frequently [4,5]. This high risk of recurrence necessitates rigorous surveillance in patients with non-muscle invasive bladder cancer. Surveillance methods include trans-urethral cystoscopy, urinary cytology, and imaging [6]. Cystoscopy can be uncomfortable for patients, and urinary cytology carries low sensitivity. These methods also incur a tremendous financial burden on the patients [7,8]. Muscle-invasive bladder cancers need aggressive treatments, including chemotherapy, radical cystectomy, radiation, immunotherapy, or a combination of these based on stages II, III, or IV and patients’ comorbidities and performance status [9]. Despite aggressive treatments, the prognosis is poor for many of these patients with muscle-invasive cancer. There is an urgent need to refine strategies for detecting recurrent localized bladder cancer and better treatment approaches for muscle-invasive bladder cancer for improved survival outcomes.

Annexin A2 (AnxA2) is a Ca^++^-dependent phospholipid binding protein, which has several functions within the cell and has been implicated in many malignant processes [10,11]. The N-terminal domain of AnxA2 contains binding sites for interaction with p11 (S100A10) and tissue plasminogen activator (t-PA), while the C-terminal end mediates binding to calcium, phospholipids, and actin filaments [12]. The structure of AnxA2 allows it to be involved in endocytosis, exocytosis, motility, fibrinolysis, linkage to the F-actin cytoskeleton, and ion channel formation [10,13,14,15,16]. AnxA2 exists as a monomer in the cytosol and a heterotetramer when linked to the cell membrane [12]. The heterotetrameric form consists of two AnxA2 monomers and two subunits of p11 and is involved in proliferation, migration, adhesion, invasion, resistance to chemotherapy, angiogenesis, plasmin generation, and ion channel conductance [11,12,13,14,17,18,19,20,21]. Several studies have shown that the AnxA2 expression is correlated with high tumor grade and stage and poor survival of cancer patients [13,22,23,24,25,26,27,28,29]. Therefore, this protein has been an attractive investigative target to evaluate its role in bladder cancer management. However, research regarding the relationship between AnxA2 and BLCA is currently lacking.

In this study, we aim to evaluate the clinical significance of AnxA2 in BLCA using the TCGA database and establish AnxA2 as a potential biomarker for BLCA patients. In addition, the expression and function of AnxA2 were evaluated by downregulating AnxA2 in bladder cancer cells. 

## 2. Materials and Methods

### 2.1. Cell Lines and Culture Conditions

The human bladder cancer cell lines RT4 and T24 were obtained from the American Type Culture Collection. Both cell lines were cultured in McCoy’s 5a medium (Hyclone Laboratories, Logan, UT, USA) supplemented with 10% fetal bovine serum (Invitrogen). The cell lines were maintained in an incubator with a humidified atmosphere of 95% air and 5% CO_2_ at 37 °C.

### 2.2. Cell Extracts and Immunoblot Analysis

Cells were washed with PBS, and total proteins were extracted using radioimmunoprecipitation assay (RIPA) lysis buffer (50 mM Tris–HCl, pH 7.5; 150 mM sodium chloride; 0.5% sodium deoxycholate; 1% Nonidet P-40; 0.1% sodium dodecyl sulfate) supplemented with phosphatase and protease inhibitor cocktail (Millipore Corporation, Burlington, MA, USA). Cell lysates were cleared by centrifugation and subjected to immunoblot analysis. Antibodies included anti-AnxA2 (BD Pharmingen #610069), anti-GAPDH (Santa Cruz Biotechnology #sc32233), and ant-β-actin (Santa Cruz Biotechnology #sc47778). The intensity of protein bands was quantified by densitometry using ImageJ software.

### 2.3. Antibody Array Assay

RayBio Human Angiogenesis Antibody Array C Series 1000 (RayBiotech, Inc., Peachtree Corners, GA, USA) was used to detect the level of 43 human proteins in cell lysates of T24 cells according to the manufacturer’s instructions. Briefly, T24 cells expressing non-targeting shRNA or AnxA2 shRNA were lysed, and the total proteins were extracted. Then, 300 μg of protein lysates were added to a blocked membrane for 4 h at room temperature. The membrane was then washed and incubated with the biotinylated antibody cocktail. The immunoblot signals were captured using the alpha-imager Fluoretech HD2. The protein signals were measured using NIH ImageJ software and analyzed using AAH-ANG-1/2 software (RayBiotech, Inc.). The results were normalized to the positive and negative controls on the array.

### 2.4. Generation of AnxA2 Knockdown in Bladder Cancer Cells

Plasmids (pGIPZ) encoding for non-silencing shRNA (Catalog number: RHS4346) and human AnxA2 shRNA (# RHS4430-200262144) were purchased from Dharmacon. The AnxA2 shRNA targeting sequence was 5ʹ- GTCTCTGTGCATTGCTGCG -3ʹ. Lentivirus was packaged in HEK293T cells using packaging plasmid VSVG and helper plasmid Δ8.9 (both from Addgene), followed by viral transduction to T24 cells with 5 µg/µL polybrene. T24 cells were then selected with puromycin (2 μg/mL, Gibco) for stable AnxA2 knockdown cells. Finally, immunoblot analysis was performed to confirm the AnxA2 knockdown in T24 cells.

### 2.5. Cell Proliferation Assay 

Cell proliferation assay was performed as described previously [30]. Briefly, bladder cancer cells were seeded in a 96-well plate at the density of 500 cells/well in McCoy’s 5a medium. After incubation, 10 μL of 3-(4,5-Dimethylthiazol-2-yl)-2,5-diphenyl-2H-tetrazolium Bromide (MTT) solution (5 mg/mL in PBS) was added to each well and incubated for additional 4 h at 37 °C. The medium was then aspirated from each well. The formazan crystals formed in the well were dissolved by adding Dimethylsulfoxide (100 μL) to each well. The absorbance of formazan at 570 nm was measured by a microplate reader (Synergy 2, BioTek Instruments, Inc., Winooski, VT, USA) at different time points.

### 2.6. Cell Migration Assay 

Cell migration assay was performed as described previously [30]. Bladder cancer cells were seeded in 6-well plates and grew into full confluency. A sterile pipette tip was used to create a clear line in each well. The growth medium was replaced with a fresh medium. The migration toward the center of the wound was determined at indicated time points.

### 2.7. Transwell Invasion Assay 

Bladder cancer cells were seeded onto the upper chamber of a transwell membrane coated with Matrigel (BD Biosciences) in a serum-free medium [31]. The lower chamber was filled with the complete serum-containing medium as a chemoattractant. The transwell plates were cultured in a CO_2_ incubator for 24 h, and then cells inside the upper chamber were removed with cotton swabs. The invaded cells that remained on the lower surface of the filter were fixed with 1% paraformaldehyde, permeabilized with methanol, and then stained with crystal violet (0.1%). The number of stained cells in six randomly selected fields of each chamber were manually counted and analyzed.

### 2.8. Immunoprecipitation

The bladder cancer cells (2.5 × 10^6^) were plated in a 100 mm Petri dish overnight in a complete medium and then replaced with a serum-free medium for another 24 h. After incubation, the serum-free medium was collected and incubated with anti-AnxA2 (BD Pharmingen #610069) antibody at 4 °C overnight. Protein A/G-PLUS agarose beads (Santa Cruz Biotechnology, Inc., Dallas, TX, USA) were then added to the medium and incubated for 2 h at 4 °C. The agarose beads were collected, washed, and resuspended in a 2× sample buffer. After boiling, the supernatant was collected and then analyzed by immunoblot analysis.

### 2.9. Elution of Cell Surface AnxA2

The confluent bladder cancer cells were washed with PBS and then incubated with Versene (0.53 mM EDTA in PBS) for 10 min at 37 °C. The Versene-wash fractions were collected and subjected to SDS-PAGE and immunoblot analysis. In addition, the Versene-wash fractions were examined for lack of cytosolic proteins by immunoblotting with an anti-3-phosphoglycerate kinase antibody.

### 2.10. Cell Surface Biotinylation

The bladder cancer cells were washed with serum-free medium and then biotinylated with sulfo-N-hydroxysulfosuccinimide (NHS) biotin (0.5 mg/mL; Cayman Chemicals) for 30 min at 37 °C in a cell culture incubator [32]. After several PBS washes, the cells were lysed in Triton-X-100 lysis buffer, and then surface-biotinylated proteins were purified by incubation with streptavidin-conjugated Sepharose (Cell signaling) overnight at 4 °C. After washing with lysis buffer, Laemmli sample buffer (2×) was added to dissociate the surface-biotinylated proteins from the beads, and they were subjected to SDS-PAGE and immunoblot analysis.

### 2.11. Plasmin Generation Assay

Chromogenic plasmin generation assay was performed in the presence of a competitive AnxA2 hexapeptide inhibitor (LCKLSL) or control peptide (LGKLSL). The bladder cancer cells were seeded equally in 12-well plates in a complete serum medium. After overnight incubation, the cell culture medium was replaced with serum-free phenol red-free medium containing either an AnxA2 hexapeptide or a control peptide or in the absence of both for an additional 4 h. The reaction was initiated by adding 100 nm Glu-plasminogen (Diapharma Group, Inc., West Chester Township, OH, USA). After 24 h, supernatant from all the wells was obtained, and after centrifugation, 100 µL of supernatant was added to a 96-well plate in triplicates. Freshly prepared 1 mM chromogenic substrate S-2403 (Diapharma Group, Inc.) was added to each well, and absorbance was measured at 410 nm (Synergy HT-BioTek) at different time intervals. The fold change in plasmin generation was calculated by normalizing the amount of plasmin generated in the untreated group.

### 2.12. RNA Expression Data for BLCA Patients

The University of California Santa Cruz browser was used to download BLCA RNA-seq data (Illumina HiSeq) from The Cancer Genome Atlas (TCGA) [33]. The Log2 transformed normalized RSEM count data for BLCA tumor samples (*n* = 409), and normal bladder samples (*n* = 19) were extracted for further analysis. The corresponding clinical information such as stage, grade, tumor histology, overall survival (OS) status, OS time (days), progression-free survival (PFS) status, PFS time (days), diseases specific survival (DSS) status, and DSS time (days) was also obtained from the BLCA-TCGA database. Survival analyses were based on Cox proportional hazard regression model to assess the effect of high vs. low AnxA2 on survival and if the effect is significant. We have reported the hazard ratio (high vs. low) and the corresponding confidence interval (CI). For each dataset, we have performed the Schoenfeld residual test for potential violation of the proportional hazards (PH) assumption, and in each case, the assumption is not violated. These analyses determined the impacts of the annexins on OS, PFS, and DSS.

### 2.13. Statistical Analysis

GraphPad Prism 8 (GraphPad Software, San Diego, CA, USA) was used for statistical analyses, and results were presented as mean ± SEM. Comparison between two groups was performed using Student’s *t*-test, while the comparison for more than two groups was performed using one-way ANOVA. Statistical significance was two-tailed and considered significant if the *p*-value was at least ≤ 0.05: (*), *p* < 0.05; (**), *p* < 0.01; (***), *p* < 0.001; (****), *p* < 0.0001.

## 3. Results

### 3.1. AnxA2 mRNA Expression Is Associated with BLCA Patients

**Figure 1:**
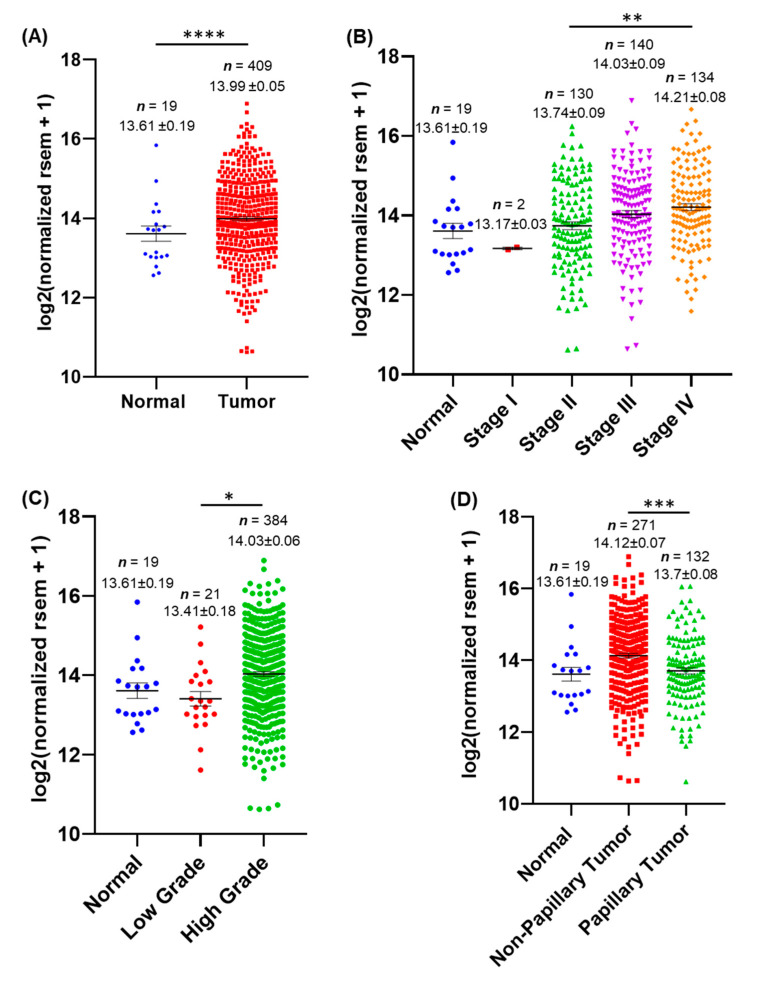
Analysis of AnxA2 mRNA expression and its clinical association in BLCA tumors from TCGA database. (**A**) Scatter plot analysis showing the AnxA2 mRNA expression in normal tissue (*n* = 19) and tumor tissue (*n* = 409) samples obtained from the TCGA-BLCA RNAseq database. Statistical analysis was performed using a two-tailed Student’s *t*-test. **** indicates *p* < 0.0001. Scatter plot analysis of AnxA2 mRNA expression in TCGA-BLCA patients based on stage (**B**), grade (**C**), and tumor histology (**D**). Statistical analyses were performed using either student’s *t*-test or one-way ANOVA followed by Tukey’s multiple comparison test. (*, *p* < 0.05; **, *p* < 0.01; ***, *p* < 0.001).

To investigate the role of AnxA2 in bladder urothelial carcinoma, we analyzed the expression of AnxA2 mRNA in normal bladder tissue samples (*n* = 19) and BLCA tissue samples (*n* = 409) from the TCGA database [34]. The expression of AnxA2 was significantly upregulated in BLCA tumor tissues compared to normal bladder tissues (*p* < 0.0001, Figure 1A). In addition, our analysis revealed that high expression of AnxA2 is significantly associated with aggressive features of BLCA. The high expression of AnxA2 was accompanied by high tumor stage and grade (Figure 1B,C). AnxA2 expression was also significantly high in non-papillary tumors (*p* < 0.001) compared to papillary tumors (Figure 1D). These observations suggest that AnxA2 expression is significantly associated with aggressive high-grade tumor phenotypes in BLCA patients.

### 3.2. High Expression of AnxA2 Is Correlated with Poor Prognosis in BLCA Patients

**Figure 2:**
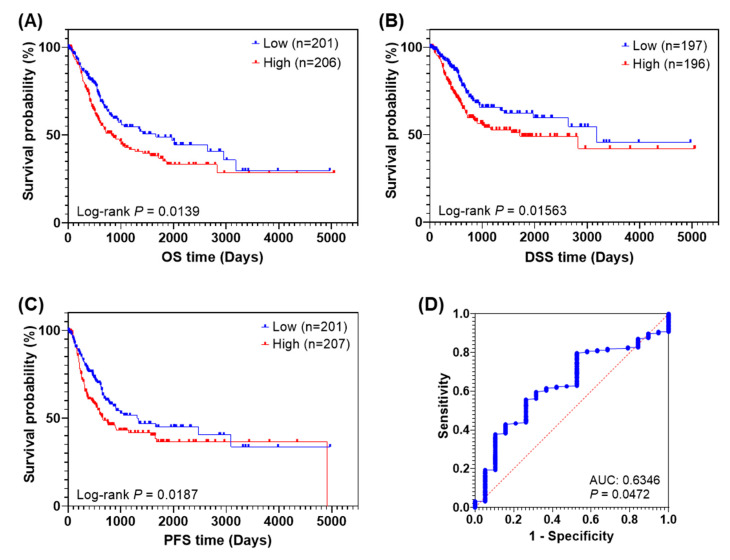
High expression of AnxA2 predicts poor prognosis in TCGA-BLCA patients. Patients in the high AnxA2 expression group had shorter OS (**A**), DSS (**B**), and PFS (**C**) compared with those in the low AnxA2 expression group. Log-rank *p*-value of <0.05 was considered statistically significant. (**D**) AnxA2 can distinguish the tumor and normal status in BLCA datasets.

We further analyzed AnxA2 mRNA expression association with survival in BLCA patients [34]. AnxA2 mRNA levels were dichotomized into low and high based on the median of logarithmized expression values. We observed a significant poor overall survival (OS) in BLCA patients with high AnxA2 mRNA expression [hazard ratio = 1.452; 95% confidence interval (CI) = 1.022−1.958, *p* = 0.0144; Figure 2A] compared to low AnxA2 mRNA expression. In addition, we also observed that high mRNA expression of AnxA2 was significantly associated with worse diseases specific survival (DSS; hazard ratio = 1.562; 95% CI = 1.085−2.249; log-rank *p* = 0.0165; Figure 2B) and worse progression-free survival (PFS; hazard ratio = 1.433; 95% CI = 1.06−1.937; log-rank *p* = 0.0193; Figure 2C) in BLCA patients. This analysis confirms that high mRNA expression of AnxA2 in tumor tissues results in poor survival in BLCA patients and suggests AnxA2 as a potential prognostic predictor of BLCA patients. Furthermore, the expression level of AnxA2 alone was able to distinguish the tumor from normal tissue, with an area under the curve (AUC) of 0.6346 ± 0.0576 (95% CI 0.5217–0.7475, *p* = 0.0472) (Figure 2D).

### 3.3. AnxA2 Expression in Bladder Cancer Cell Lines

**Figure 3:**
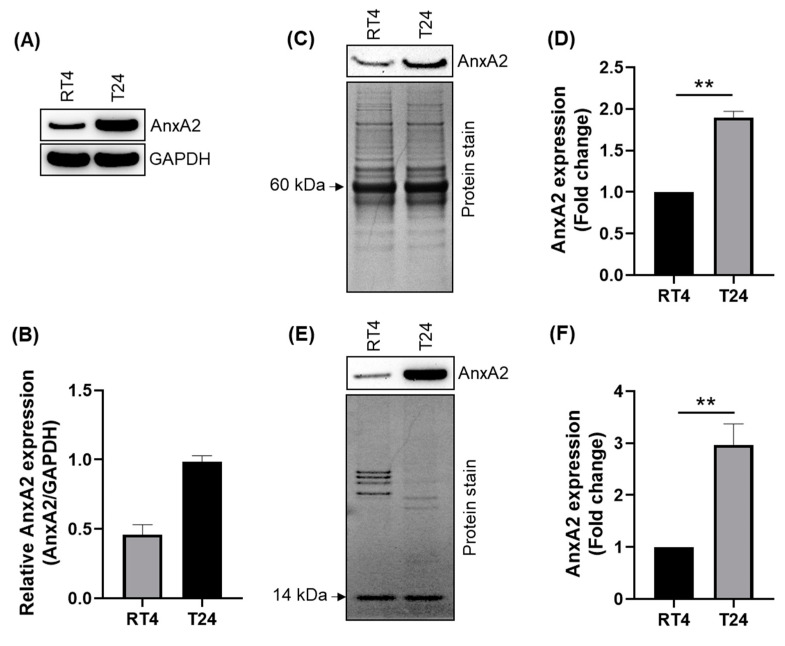
AnxA2 expression in human bladder cancer cell lines. (**A**) Endogenous AnxA2 protein was analyzed by immunoblot analysis in RT4 and T24 bladder cancer cell lines. Glyceraldehyde-3-phosphate dehydrogenase (GAPDH) was used as a loading control. (**B**) The intensity of AnxA2 was determined by densitometry and normalized by loading control. (**C**) Cell surface expression of AnxA2 in bladder cancer cells was analyzed by a membrane wash experiment. Cells were incubated with Versene for 5 min, and then the supernatant was collected by centrifugation for immunoblot analysis. SDS-PAGE was performed with identical protein concentrations to normalize for loading, and the gel was stained with Coomassie Blue. (**D**) Densitometric analysis was performed to compare the fold change in AnxA2 expression. (**E**) The cell surface biotinylation was performed as described in the material and methods. The extracts were immunoblotted with an anti-AnxA2 antibody. Coomassie Blue staining was performed for loading control. A ∼14-kD, Coomassie-stained band, whose levels seemed invariant in both cell lines, was used to account for differences in protein loading. (**F**) The intensity of AnxA2 was performed by densitometry, and fold change in AnxA2 expression was determined. **, *p* < 0.01. Uncropped Western Blots of (**A**,**C**,**E**) are available in File S1.

The BLCA RNAseq analysis demonstrated that AnxA2 mRNA expression was significantly elevated in high-grade tumors compared to low-grade tumors in BLCA patients. therefore, the expression of AnxA2 protein was compared by immunoblot analysis in lysates prepared from RT4 and T24 bladder cancer cell lines, which were derived from a grade I urothelial carcinoma (also called papilloma) patient [35] and poorly differentiated (grade III) bladder carcinoma with metastatic profile [36], respectively. The immunoblot presented in Figure 3A,B showed that AnxA2 protein expression was significantly higher in T24 bladder cancer cells than in RT4 cells. Our results further confirm that high expression of AnxA2 is significantly associated with the aggressive phenotype of bladder cancer. In addition, we have further examined whether increased expression of AnxA2 in bladder cancer mobilizes it to the outer membrane. To elute cell surface AnxA2, we used Versene (0.53 mM EDTA in PBS) solution to wash the cells. Because AnxA2 is a Ca^++^-dependent phospholipid-binding protein, the AnxA2 protein should be dissociated from the cell surface after Versene wash. Therefore, we collected the Versene-wash fractions and analyzed them with immunoblotting. The blots in Figure 3C,D show that a large pool of AnxA2 is present in the Versene wash fraction of T24 cells compared to RT4 cells. We also analyzed the cell surface levels of AnxA2 in bladder cancer cells by conjugating all cell surface proteins with membrane-impermeable biotin and immobilizing them with streptavidin-linked beads. Immunoblot analysis of biotin-conjugated and streptavidin-immobilized cell surface extracts revealed that AnxA2 expression at the cell surface of T24 cells was 3-fold higher compared to RT4 cells (Figure 3E,F). Coomassie staining was used as protein loading controls for both Versene-wash fractions and cell surface biotinylated extracts. These results suggest that high cell surface expression of AnxA2 is associated with aggressive phenotypes of bladder cancer cells.

### 3.4. High Expression of AnxA2 at the Cell Surface of Bladder Cancer Cells Promotes Plasmin Generation

**Figure 4:**
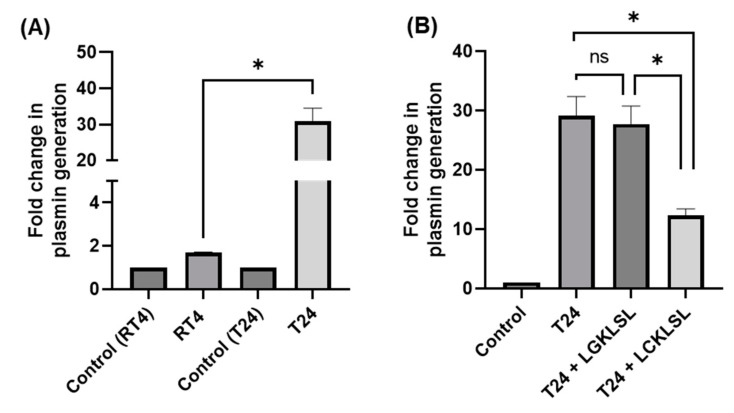
AnxA2-mediated plasmin generation. (**A**) Chromogenic plasmin generation assay was performed in RT4 and T24 bladder cancer cell lines as described in the materials and methods. To serve as a control, Glu-plasminogen was omitted from the reaction mixture. The fold increase in plasmin generation was determined. (**B**) Chromogenic plasmin generation assay was performed in T24 in the presence of either AnxA2 inhibitory hexapeptide (LCKLSL; 1 mM) or control peptide (LGKLSL; 1 mM). The fold change in plasmin generation was obtained. The data are means ±SEM (*n* = 3). ^ns^, non-significant; *, *p* < 0.05.

AnxA2-mediated plasmin generation assay was performed to examine whether an increased cell surface pool of AnxA2 in bladder cancer cells is associated with an extensive production of plasmin. First, we compared the biochemical conversion of plasminogen to plasmin in RT4 and T24 cell lines (Figure 4A). We found that plasmin production is approximately 18-fold higher in T24 cells than in RT4 cells. To further confirm that the high production of plasmin in T24 cells is due to the increased cell surface pool of AnxA2, we treated the cells with a competitive hexapeptide inhibitor (LCKLSL) of tPA, which binds to AnxA2 or control peptide (LGKLSL) which does not possess any plasmin inhibitory effects [37,38]. As shown in Figure 4B, treating T24 cells with LCKLSL peptide resulted in a 2.3-fold reduction in plasmin generation compared with the control peptide, LGKLSL. These results suggest that the increased cell surface levels of AnxA2 in T24 cells directly contribute to the high production of AnxA2-mediated cell surface generation of plasmin.

### 3.5. AnxA2 Secretion from Bladder Cancer Cells

**Figure 5:**
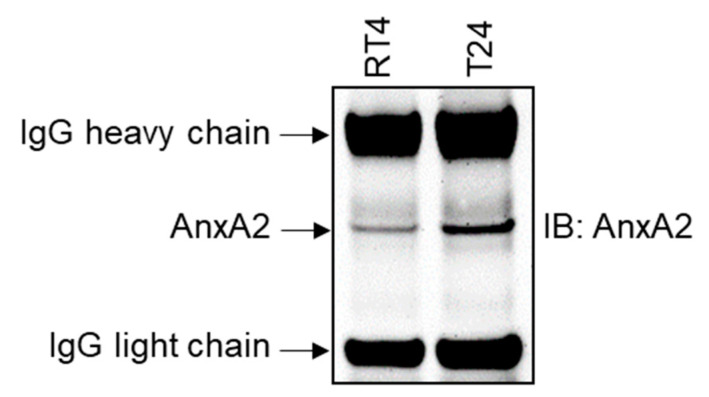
AnxA2 secretion from bladder cancer cell lines. The RT4 or T24 cells (2.5 × 10^6^) were plated in a 100 mm Petri dish overnight and then replaced with a serum-free medium. AnxA2 protein was immunoprecipitated from the serum-free medium after 24 h incubation using an anti-AnxA2 antibody (BD Biosciences #610069). The amount of secreted AnxA2 in the medium was analyzed by immunoblotting using an anti-AnxA2 antibody. Uncropped Western Blot is available in File S1.

Although AnxA2 does not have signals for secretion, other investigators reported it as a secretory protein [22,25,27,39,40,41]. Therefore, we examined the secretion of AnxA2 in the condition media of RT4 and T24 bladder cancer cells. Secreted AnxA2 protein was immunoprecipitated from the serum-free condition media of RT4 and T24 cells using an anti-AnxA2 antibody. On immunoblotting with anti-AnxA2 antibody, high expression of AnxA2 was predominantly observed in conditioned media of T24 cells. In contrast, deficient AnxA2 expression was detected in RT4 cells (Figure 5), indicating that the cells derived from high-grade tumors secrete more AnxA2 than those derived from low-grade tumors.

### 3.6. Depletion of AnxA2 Inhibits the Proliferation, Migration, and Invasion of Bladder Cancer Cells

**Figure 6:**
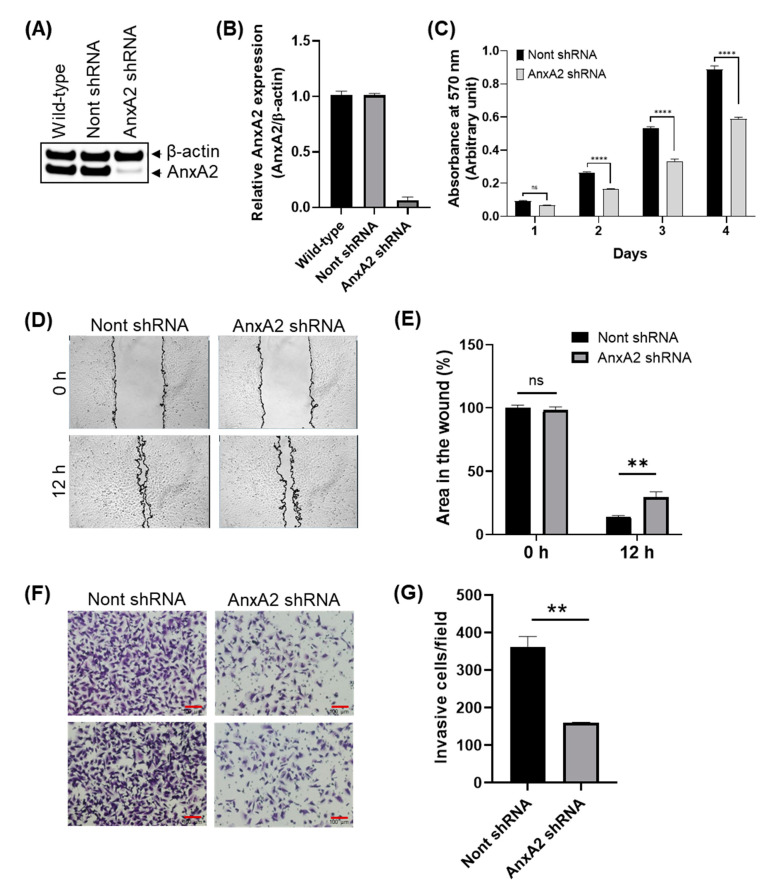
Effect of AnxA2 depletion on proliferation, migration, and invasion in T24 bladder cancer cells. (**A**) AnxA2 was knockdown in human bladder cancer cells using shRNA. Immunoblot analysis showed that AnxA2 expression was efficiently knockdown by AnxA2 shRNA in T24 cells. β-Actin was used as a loading control. (**B**) The intensity of AnxA2 bands was determined by densitometry and normalized by loading control. The bar graph represents the fold decrease in AnxA2 in shRNA knockdown T24 cells compared with control T24 cells. (**C**) bladder cancer cells were seeded in 96-well plate at the density of 500 cells/well in McCoy’s 5a medium for incubated for different time periods. MTT analysis was performed as described in material and methods to assess the cell proliferation. (**D**). Representative images from wound healing migration assays performed with the AnxA2 knockdown T24 cells. (**E**) Bar graph showing the percentage of wound closure after 12 h of wound formation. (**F**) Representative images from transwell invasion assay performed with the AnxA2 knockdown T24 cells. (**G**) Bar graph showing the number of invaded cells. Data are presented as mean ± SEM, ^ns^, non-significant; **, *p* < 0.01; ****, *p* < 0.0001. Uncropped Western Blot of (A) is available in File S1.

To elucidate the biological functions of AnxA2, we down-regulated the expression of AnxA2 in the bladder cancer T24 cells. The depletion of AnxA2 was detected by immunoblot analysis. We found that the stable transfection of AnxA2 shRNA effectively reduces the expression of AnxA2 in T24 cells (Figure 6A,B). The expression level of AnxA2 was only 9% of the cells transfected with non-targeting shRNA, indicating that AnxA2 shRNA significantly reduced the AnxA2 levels in T24 cells. On this basis, we explore the effects of AnxA2 on bladder cancer cell proliferation by performing the MTT assay. The results revealed that AnxA2 knockdown significantly impaired the proliferation of T24 cells. The cell proliferation was considerably lower in AnxA2-depleted cells compared to the non-targeting shRNA-transfected cells after 2, 3, and 4 days of time intervals (*p* < 0.0001) (Figure 6C). The role of AnxA2 in T24 bladder cancer cell migration was assessed using the wound healing assay. Significant closure of the scratch wound was observed in the T24 cells transfected with non-targeting shRNA after 12 h. However, scratch wound closure was significantly inhibited in AnxA2-depleted T24 cells (*p* < 0.01) compared to non-targeting shRNA transfected cells (Figure 6D,E). Transwell assays were performed to examine the role of AnxA2 in bladder cancer cell invasion. The number of T24 cells that had migrated through to the bottom of the transwell membrane was significantly lower in the AnxA2-depleted T24 cells compared to the T24 cells transfected with non-targeting shRNA (*p* < 0.01; Figure 6F,G). These results have demonstrated that AnxA2 plays a crucial role in bladder cancer cell proliferation, migration, and invasion.

### 3.7. Depletion of AnxA2 Downregulates Pro-Angiogenic Factors in Bladder Cancer Cells

**Figure 7:**
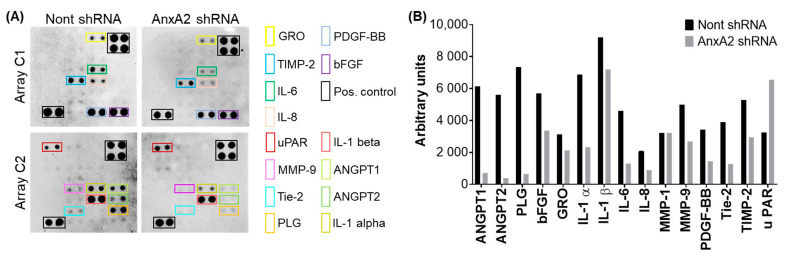
The differential expression of angiogenic growth factors and cytokines in T24 cells expressing non-targeting shRNA or AnxA2 shRNA. (**A**) The human angiogenesis antibody array membrane, onto which 43 angiogenic proteins are blotted in duplicates, was incubated with cell lysates of T24 cells expressing non-targeting shRNA or AnxA2 shRNA. The proteins showing expression in T24 cells were highlighted in rectangles. (**B**) Arrays were analyzed using densitometry and normalized to the internal control. The densitometric arbitrary units indicate the increase or decrease of the intensity of proteins in AnxA2-depleted T24 cells compared to control cells. Uncropped Western Blot of (A) is available in File S1.

Depletion of AnxA2 inhibits the migration and invasion of a highly metastatic T24 cell line. To identify the migration and invasion-associated factors whose expression is regulated by AnxA2, we performed angiogenic factors and cytokines antibody array. We found that the knockdown of AnxA2 in T24 cells decreased the expression of several growth factors and cytokines, including basic fibroblast growth factor (bFGF), platelet-derived growth factor-BB (PDGF-BB), angiopoietin-1 (ANGPT1), angiopoietin-2 (ANGPT2), angiostatin (PLG), growth-related oncogene (GRO), interleukin-1 alpha (IL-1 alpha), interleukin-1 beta (IL-1 beta), interleukin-6 (IL-6), interleukin-8 (IL-8), matrix metalloproteinase-9 (MMP-9), tunica intima endothelial receptor tyrosine kinase 2 (Tie-2) and tissue inhibitor of metalloproteinases 2 (TIMP-2) (Figure 7A,B). However, the expression of urokinase plasminogen activator surface receptor (uPAR) was found to be upregulated in AnxA2 knockdown T24 bladder cancer cells. These results indicate that depletion of AnxA2 inhibited the migration and invasion of bladder cancer cells via the blockade of multiple angiogenic proteins and cytokines.

## 4. Discussion

Bladder urothelial carcinoma is a highly heterogeneous disease with varying clinical outcomes. Patients with the same tumor stage may have different survival outcomes [2,3,4,5,42]. The survival rates correlate directly with the cancer cell proliferation, migration, and invasion of bladder cancer. Thus, it is essential to identify molecular markers to detect bladder cancer early before cancer cells become invasive and metastatic. Annexins display different expression patterns in bladder cancer and play an essential role in the pathogenesis by triggering various downstream signaling pathways and functional regulatory effects [19,20,43,44,45,46,47,48]. Increasing evidence indicates that AnxA2 is upregulated in several types of human cancer [13,17,21,22,24,26,27,28,29]. Annexin A2 is a potential biomarker for predicting drug resistance and recurrence of bladder cancers [19,20]. The TCGA data suggests that AnxA2 was significantly overexpressed in BLCA tumors. A high abundance of AnxA2 was noticeably associated with high grade and stage and aggressive histologic grade bladder cancer and revealed poor prognosis in bladder cancer patients. We found that the AnxA2 protein was upregulated in bladder cancer cells derived from high-grade tumors and also associated with higher secretion in the media. In vitro assays demonstrated that depletion of AnxA2 significantly inhibited the proliferation, metastasis, and invasion of bladder cancer cells by downregulating the expression of proangiogenic proteins and cytokines. Our results provide a strong case for AnxA2 as a potential prognostic predictor with poor clinical outcomes for bladder cancer patients.

Upregulation of AnxA2 has been observed in gastric cancer, endometrial cancer, pancreatic ductal adenocarcinoma, hepatocellular carcinoma, breast cancer, kidney renal clear cell carcinoma, high-grade prostate cancer, and glioblastoma. It has a significant correlation with advanced stage and unfavorable prognosis [13,17,21,22,24,26,27,28,29]. Previous studies suggested that AnxA2 was upregulated in bladder urothelial carcinoma tumor tissues and associated with lymph node metastasis and distant metastasis [19,20]. Based on these findings, we conducted a bioinformatics analysis to explore the expression of AnxA2 in the BLCA-TCGA database. Our analysis confirmed that a high expression level of AnxA2 was noticeably associated with high tumor stage and grade and an unfavorable prognosis of bladder cancer. Furthermore, the high expression of AnxA2 was significantly observed in non-papillary carcinoma, which is usually high-grade and invasive, compared to papillary carcinoma, which is usually low-grade and non-invasive [49,50]. Furthermore, Kaplan–Meier curves analysis indicated that AnxA2 was a prognostic factor for BLCA. Together, these results further established that high mRNA expression of AnxA2 might contribute to the tumorigenesis of BLCA and be an essential risk factor for the poor clinical outcome of bladder cancer patients.

AnxA2 is primarily present in the cytoplasm and cell membrane of the cells, and a small fraction is also present in the nucleus [12,13,15,21]. AnxA2 exists either as a monomer or a heterotetramer complex formed by two of p11 and two AnxA2 monomers [11]. Upon translocation to the outer cell membrane, the AnxA2 heterotetramer complex serves as a receptor for tPA, converts plasminogen into plasmin, and promotes angiogenesis. Our study indicated that cell surface expression of AnxA2 is very high in T24 cells derived from a high-grade tumor. In addition, our results also showed that high cell surface expression of AnxA2 in T24 cells produces a large amount of plasmin which plays an important role in angiogenesis and metastasis of high-grade bladder urothelial carcinoma [51,52]. Blocking the activity of the AnxA2 using LCKLSL hexapeptide can have potential therapeutic implications on inhibiting the pathological processes associated with enhanced plasmin generation in high-grade aggressive BLCA [18,37,38]. Furthermore, Lu et al. identified secretory AnxA2 as a potential urine biomarker for urothelial carcinoma of the upper urinary tract [53]. Our results are consistent with these findings in urothelial carcinoma of the bladder. The high secretion of AnxA2 from T24 cells was significantly observed with aggressive subtypes of bladder cancer.

Several studies have reported that overexpression of AnxA2 activates several signaling pathways involved in tumor proliferation, invasion, migration, angiogenesis, and metastasis [10,13,14,21]. Depletion of AnxA2 is known to be involved in the downregulation of proangiogenic proteins, including vascular endothelial growth factors such as VEGF-C, VEGF-R2, matrix metalloproteinases such as MMP-2, MMP-9, MT1-MMP, cytokine such as IL-6, and also the tissue inhibitor of metalloproteinase-2 (TIMP-2) [54,55,56]. Consistent with previous studies, we also found that AnxA2 knockdown significantly inhibited bladder cancer cell proliferation, migration, and invasion, along with remarkably decreased expression of proangiogenic proteins such as PDGF-BB, ANGPT1, ANGPT2, Tie-2, bFGF, GRO, IL-6, IL-8, and MMP-9. In addition, the high expression of ANGPT1, bFGF, MMP-9, and Tie-2 has been reported as an important prognostic factor for the high-risk group of bladder cancer patients [57,58,59,60].

However, our study has some potential limitations. First, we must collect the tumor tissue samples from bladder urothelial carcinoma patients and relevant prognoses to confirm further the AnxA2 expression and its association with clinical outcomes. Secondly, we must determine how AnxA2 promotes bladder cancer cell proliferation, migration, and invasion. These limitations will be addressed in future studies.

## 5. Conclusions

In conclusion, our results demonstrated that high expression of AnxA2 promotes proliferation, migration, and invasion in bladder cancer cells, and AnxA2 mRNA expression was significantly associated with the poor prognosis of BLCA patients. To our knowledge, this is the first report that demonstrated the biological function of AnxA2 in Bladder urothelial carcinoma. Our study provides evidence that AnxA2 might be a potential therapeutic target and prognostic marker for Bladder Urothelial Carcinoma.

## Data Availability

The data supporting this study’s findings are available from the corresponding author upon reasonable request.

## Supplementary Materials

The following supporting information can be downloaded at: https://www.mdpi.com/article/10.3390/cancers14225664/s1, File S1: Original blots.
