# Supplementary material for: Higher Expression of Annexin A2 in Metastatic Bladder Urothelial Carcinoma Promotes Migration and Invasion
Source: Cancers (Basel). 2022 Nov 18;14(22):5664. doi: 10.3390/cancers14225664 (PMC9688257; doi:10.3390/cancers14225664)
Supplement: Supplementary file 1 [file cancers-14-05664-s001.zip › cancers-2010597-Supplementary File S1.Original blots .pdf]

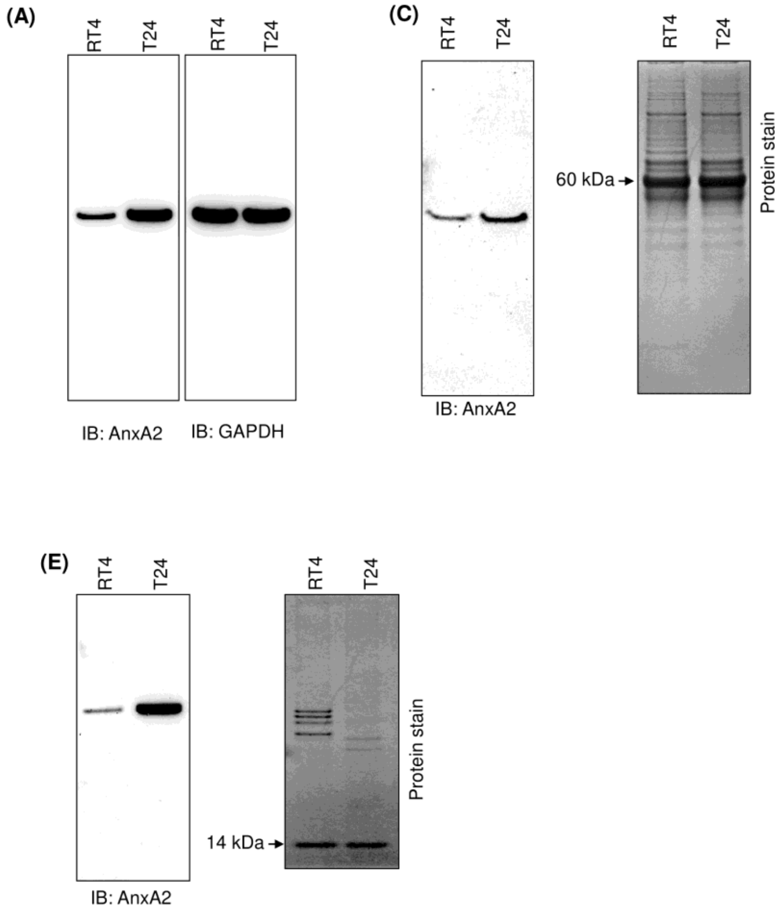

Figure S1. Original blots of Figure 3.

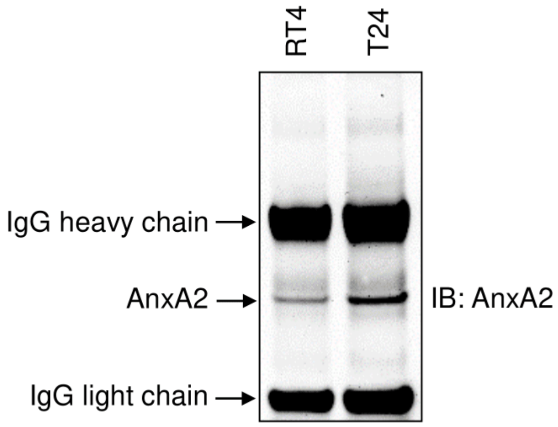

Figure S2. Original blots of Figure 5.

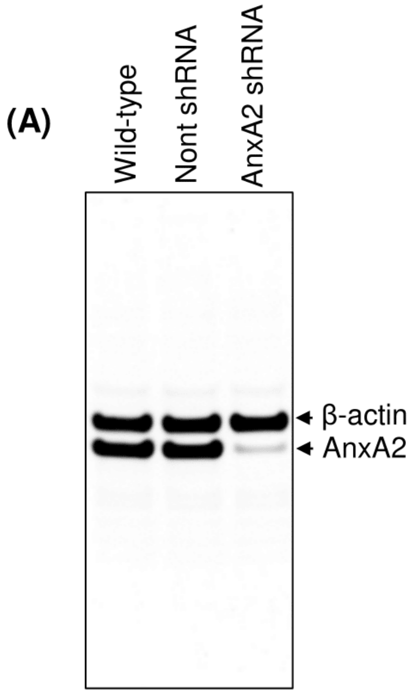

Figure S3. Original blots of Figure 6.

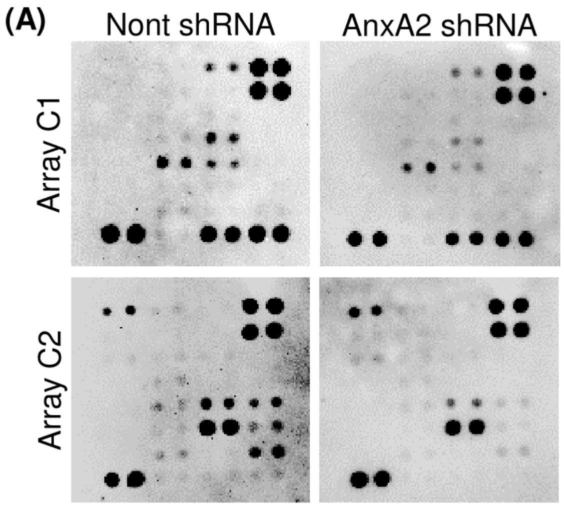

Figure S4. Original blots of Figure 7.
